# Comorbidity of obstructive sleep apnea and narcolepsy: A challenging diagnosis and complex management

**DOI:** 10.1016/j.sleepx.2024.100126

**Published:** 2024-09-18

**Authors:** Silvia Miano, Leila Kheirandish-Gozal, Marco De Pieri

**Affiliations:** a Sleep Medicine Unit, Neurocenter of Southern Switzerland, Civic Hospital, EOC, Faculty of Biomedical Sciences, Università della Svizzera Italiana, 6900, Lugano, Switzerland; b Department of Neurology, Pediatric Sleep Medicine, University of Missouri, United States; c Division of Adult Psychiatry, Department of Psychiatry, University Hospitals of Geneva, 2 Chemin du Petit-Bel-Air, CH-1226, Thonex, Switzerland

**Keywords:** Narcolepsy, Sleep apnea, Sleepiness, Treatment, Epidemiology, Children

## Abstract

**Introduction:**

Narcolepsy and obstructive sleep apnea syndrome (OSA) are relevant causes of excessive daytime sleepiness (EDS); although different for etiopathogenesis and symptoms, differential diagnosis is sometimes difficult, and guidelines are lacking concerning their management when coexisting in a same patient.

**Methods:**

A narrative review of the literature was realized including PubMed, Scopus and Embase, aimed to regroup studies and case reports evaluating epidemiology, clinical and instrumental features and treatment of patients presenting comorbid NT1 and OSA. Moreover, a snowball search on the pathophysiology underpinnings of the association of the two disorder was realized.

**Results:**

For adults, the prevalence of OSA in NT1 ranged from 24.8 % to 51.4 %. No studies were found concerning the treatment of EDS in double-diagnosis patients, but only case reports; these latter and the experience on patients with either NT or OSA suggest that modafinil, methylphenidate, pitolisant and solriamfetol are effective.

**Discussion:**

Adults with NT1 showed a higher prevalence of OSA compared to the general population, but the reach of the results reviewed here is limited by the retrospective design of most of the studies and by the inhomogeneous utilization of diagnostic criteria. The association with OSA is likely to be explained by the involvement of orexin in hypercapnic-hypoxic responses: a deficit of orexin may promote obstructive events during sleep. Open questions warrant further investigation, especially orexin's involvement in other sleep disorders associated with EDS, and the more appropriate treatment for the OSA-narcolepsy comorbidity.

## Highlights

• Differentiate narcolepsy and obstructive sleep apneas syndrome (OSA) is difficult.
• Prevalence of OSA in narcolepsy seems increased compared to the general population.
• Orexin could be involved in hypercapnic-hypoxic responses, thus preventing apneas.
• No clear indications on the management of comorbid narcolepsy and OSA.

## 1 Introduction

Obstructive sleep apnea (OSA) is a widespread disorder influenced by multiple factors, encompassing anatomical constriction and reduced muscle tone essential for preserving upper airway patency. The chronic sleep fragmentation stemming from recurrent intermittent hypoxia and cortical arousal manifests as excessive daytime sleepiness (EDS) [1]. The pathophysiology of OSA also relies on a chronic low-grade inflammation: sleep fragmentation related to sleep apnea and cyclic hypoxia are considered to induce a dysregulation of interleukin 6 (IL 6) and tumor necrosis factor alfa (TNF-alfa) [2].

Curiously, research studies show patients with OSA experience persistent sleepiness despite adequate treatment with nasal continuous positive airway pressure (CPAP) suggesting the possibility of a missing link.

Narcolepsy is a lifelong non-progressive neurological disease characterized by dysregulation of the sleep-wake cycle with multiple intrusions of rapid eye movement (REM) sleep. The classic tetrad of symptoms associated with narcolepsy; EDS, cataplexy, sleep paralysis, and hypnagogic hallucinations are often accompanied by disrupted nocturnal sleep. The International Classification of Sleep Disorders (ICSD-3) characterizes narcolepsy type 1 (NT1) by the presence of cataplexy and distinguishes type 2 by the absence of this phenomenon and the assessment of orexin levels [3].

The etiopathogenesis of NT1 seems to rely on the T-cell mediated destruction of hypocretin cells in the lateral hypothalamus, a process that could be promoted by a molecular mimicry between infectious or vaccinal and endogenous epitopes [[4], [5], [6]]; the infections with H1N1 influenza and streptococcus pyogenes, and influenza vaccination have been described as a trigger for NT1 [[7], [8], [9]].

Investigations into the association between orexin levels and EDS in the general population using the Epworth Sleepiness Scale (ESS) yielded inconclusive results, with no association found with the human leucocyte antigen (HLA) DQB10602 allele, but spectral analysis revealing differences in sleep propensity among individuals with OSA who were HLA-DQB10602-positive and HLA-DQB1∗0602-negative [10].

For both OSA and NT1 an association with genetic factors was described. For NT1, stronger evidences are related to the HLA system, mainly involving the haplotype DQB1∗06:02, but also, less commonly, other alleles, such as DQB1∗03:02, DQB1∗03:01, DQB1∗06:03, DQB1∗05:01 and DQB1∗06:01. The other main genes involved are the T-cell receptor alfa chain gene, the purinergic receptor subtype 2Y11, catepsin H and the tumor necrosis factor ligand superfamily member 4 (TNFSF4) [11].

For OSA, replicated candidate gene findings are an association with the −308G/A polymorphism of the TNF-alfa gene, with the rs1409986 polymorphism in the Prostaglandin E2 receptor EP3 subtype (PTGER3) and the rs7030789 polymorphism of the Lysophosphatidic acid receptor 1 (LPAR1) [12]. Moreover, genome-wide association studies (GWAS) produced non-replicated findings [13,14].

While a heightened incidence of OSA has been suggested in adults diagnosed with NT1, the intricate mechanisms linking narcolepsy and OSA, as well as the impact of OSA on daytime sleepiness, remain areas of ongoing investigation and have not been fully elucidated [15]. Beyond the distinctive features of cataplexy and hypnagogic hallucinations, both narcolepsy and OSA manifest with EDS as a predominant clinical characteristic. Sleep fragmentation, disruptions in sleep continuity, fatigue, and weight gain are additional shared clinical manifestations as documented by Drake [16] and Paudel et al. [17].

From a historical perspective, the initial documentation of comorbid sleep-disordered breathing focused on the observation of central sleep apnea in patients diagnosed with narcolepsy. Coccagna and colleagues [18] described hypersomniac-hypoventilation syndrome characterized by central periodic breathing at the onset of sleep, with spontaneous remission of sleep-disordered breathing over time. The authors posited a potential involvement of the brainstem in explaining the abnormalities in sleep-related breathing, obesity, and hypersomnia. Building on this foundation, Guilleminault and collaborators [19] conducted a study on two narcolepsy-cataplexy patients, uncovering central apnea episodes lasting 20–90 s during stages N1, N2, and REM. Additionally, Kales and colleagues [20] reported a case of a narcoleptic patient intolerant to methylphenidate due to cardiac dysrhythmias, successfully managed with propranolol, which alleviated EDS, cataplexy, and sleep apnea characterized by 30–70 central apneic episodes per hour, and in a subsequent case, from an autopsy of a 17-year-old obese boy with narcolepsy symptoms, Drake [16] presented findings attributing his demise to a potential overlap of narcolepsy and OSA as evidenced by cardiomegaly and right ventricular enlargement. During the same year, Kales and colleagues [21] investigated the prevalence of sleep apnea in a cohort of 50 narcoleptic patients with cataplexy, revealing only one severe case of sleep apnea. Subsequently, Chokroverty and colleagues [22] examined 16 narcoleptic patients through polysomnographic recordings and multiple sleep latency tests (MSLT), identifying a spectrum of apnea types, including purely central, both central and obstructive, and mixed apnea. Importantly, neither obesity nor advancing age emerged as significant factors in the development of sleep apnea within this limited case series.

These early reports collectively underscored the association between sleep-disordered breathing, predominantly characterized by a central pattern, and various narcoleptic phenotypes. Confirming a diagnosis of narcolepsy can indeed pose challenges. In the absence of clear indicators such as cataplexy and the presence of orexin deficiency or specific HLA markers, the potential for misdiagnosis of narcolepsy in comorbidity with SDB may not be disregarded. It is noteworthy that MSLT in untreated OSA might yield false-positive results for narcolepsy due to reduced sleep latency, especially when patients report atypical cataplexy [17]. The comorbidity between OSA and narcolepsy may exhibit bidirectional influences. In a noteworthy case [23], the complexities of diagnosis became evident when managing the concurrent presence of narcolepsy and OSA. In this scenario, narcolepsy was identified in an individual undergoing CPAP treatment for OSA, after the discovery of a low orexin level, assessed for persisting of EDS. Notably, the MSLT failed to reveal sleep onset REMs (SOREMPs). The absence of REM sleep during daytime testing was attributed to apneic events, potentially compromising the sensitivity and specificity of MSLT, particularly in cases marked by a high frequency of apneic events. Aguilar and colleagues [24], described the case of a 28-year-old who exhibited severe mandibular retrognathia, EDS, snoring, apnea, a history of cocaine abuse, and severe OSA. The initial PSG indicated an AHI of 25.9/hr, but the patient initially declined CPAP therapy; some months later he accepted this treatment, albeit without improvement in EDS. During a follow-up clinical visit, the patient disclosed a history of sleep attacks, sleep paralysis, and cataplexy throughout his life. A PSG recorded with CPAP was succeeded by an MSLT, which uncovered a notably low sleep latency (2 min) and three SOREMPs. The presence of the HLA-DQB1∗0602 allele and an undetectable cerebral spinal fluid (CSF) orexin level ultimately confirmed the diagnosis of narcolepsy.

Factors such as obesity and those associated with orexin dysfunction could heighten the susceptibility of narcoleptic patients to OSA. Conversely, OSA may induce a secondary narcoleptic-like condition through mechanisms involving chronic intermittent hypoxia and heightened arousal during sleep, thereby causing direct damage to orexinergic neurons [25].

Individuals presenting at sleep centers with EDS, alongside comorbid OSA, may be diagnosed solely with OSA, potentially leading to an oversight of the coexisting presence of narcolepsy.

**Fig. 1:**
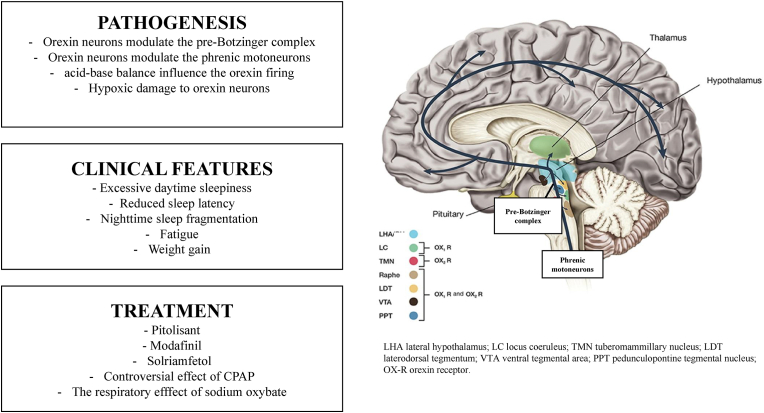
The overlap between OSA and NT1.

A narrative review of the literature was conducted using PubMed, Scopus, and Embase, to evaluate epidemiology, clinical and instrumental features, and treatment of patients presenting comorbid NT1 and OSA. Furthermore, a snowball search on the pathophysiological underpinnings of the association of the two disorders was conducted. Fig. 1 outlines the content of the present review.

## 2 Prevalence

The estimated prevalence of OSA in the general population is reported to range between 15 and 30 % in males and 10–15 % in females, as substantiated by various studies and secondary analyses [26,27]. The prevalence of NT1 is estimated at 0.02–0.05 % across Western countries [11,28]. According to literature data, the prevalence of OSA in individuals with NT1 has been documented within the range of 24.8 %–51.4 % (see Table 1).

**Table 1:** Clinical features and multiple sleep latency test (MSLT).

Reference	N	OSA (%)	Age (Yrs)	Sex (F)	CSF Orexin (pmol/L)	Cataplexy (yes)	HLA DQB1∗ 0602	ESS	BMI (kg/m^2^)	MSLT		
										*SL (min)*	*REM latency (min)*	*SOREMPs (N)*
App 1990	46	28	Na	34.5 %	Na	46.5 %	Na	Na	Na	Na		
Sansa 2010	133	24.8	38.6 ± 16.4	34 %	Na	78 %	Na	17.7 ± 4.3	23.9 ±4.7	2.1± 1.8	4.8 ± 2.7	3.2 ± 1.4
Pataka 2012	102	28.5	45.7 ± 16.5	60 %	Na	74.5 %	84.3 %	18.3 ± 4	27.9 ±5.8	4.5± 3.2	3.9 ± 3.2	2.7 ± 1.1
Frauscher 2013	100	24	39 [16–78]	44 %	Na	87 %	93 %	18 [10–24]	26.2 [18.2–43]	10.7± 3.2	Na	4 (0–5)
Jennum 2013	757	Na	40.3 ± 35.8	53.6 %	Na	Na	Na	Na	Na	Na		
Nevsimalova 2013	117	30.77	47.8 ± 17.2	Na	Na	79.5 %	84.7 %	18.0 ± 3.4	Na	2.7± 2.1	Na	3.4 ± 1.1
Black 2017	9312	51.4	46.1 ± 13.3	59.2 %	Na	20.3 %	Na	Na	Na	Na		
Hoshino 2019	141	39.4	30.7 ± 12.7	31.9 %	Na	26 %	Na	14.8 ± 5.1	23.3 ±4.3	2.3± 1.6	4.9 ± 2.7	3.2 ± 1.1
Filardi 2020	38	13.6	11.66 ± 3.77	34.2 %	32.70 ± 43.26 pg	Na	100 %	15.53 ± 3.53	24.39 ±5.09	2.70 ±2.35	Na	4.58 ± 0.83

Data expressed as mean ± standard deviation or median [range]; N number of subjects with narcolepsy; AHI apnea-hypopnea index; BMI body mass index; CSF cerebrospinal fluid; ESS Epworth sleepiness scale; SL sleep latency; SOREMP sleep onset REM period. ∗the study only included OSA patients undergoing adenotonsillectomy for OSA.

In a research study involving 1000 patients exhibiting EDS, 46 individuals were diagnosed with Narcolepsy NT1 and OSA. Notably, the diagnosis of narcolepsy was conclusively established after the successful resolution of OSA through CPAP therapy. Corroborating the findings, the severity of OSA exhibited a positive correlation with body mass index (BMI). However, intriguingly, the severity of EDS, as assessed through the MSLT, did not demonstrate improvement with CPAP intervention [29]. In a subsequent study, a cohort of 133 consecutive narcoleptic patients was systematically assessed between the years 1991 and 2007. Among these individuals, 33 patients were identified as having comorbid OSA with an Apnea-Hypopnea Index (AHI) ≥ 10 events per hour). Notably, this subgroup exhibited characteristics such as older age, male gender, and a higher BMI [30].

In a retrospective chart review study of 102 narcoleptic patients, twenty-nine of them (28.5 %) had a comorbid OSA confirmed by polysomnography. Treatment with CPAP demonstrated a statistically significant improvement in Epworth Sleepiness Scale (ESS) scores over one year of follow-up [31]. Another study involved 100 consecutive subjects diagnosed with NT1, and it was revealed that 24 % of the participants exhibited comorbid OSA. The severity of OSA in this cohort varied, encompassing cases ranging from mild to severe [32]. Similarly, findings from another study by Nevsimalova et al. [33] highlighted that severe OSA, characterized by an AHI exceeding 30/hr, was exclusively observed in narcoleptic patients aged 40 and above. In a retrospective population study in the United States conducted by Black and colleagues [34], the prevalence of comorbid OSA in narcoleptic patients was reported to be 51.4 %. In a controlled retrospective national study, which specifically focused on NT1, sleep apneas were identified as significant comorbidity, both occurring before and persisting after the diagnosis of NT1, with a 44.5 [13.1–151.3] odds ratio [35]. A Japanese study in Japan involved 141 adults with narcolepsy, revealing that 39.7 % of them had comorbid OSA [36]. Individuals with OSA in this cohort were characterized by advanced age, higher BMI, an increased prevalence of cataplexy, lower sleep efficiency, and a higher arousal index.

Few studies have investigated the comorbidity between narcolepsy and OSA in children. Filardi and colleagues [37] observed that the prevalence of OSA in narcoleptic children was 13.6 %, reaching its peak at the same age as observed in children without narcolepsy. A case report described a 4-year-old girl with moderate OSA, where the diagnosis of comorbid narcolepsy was established following adenotonsillectomy [38]. Similarly, in a case reported by Almbaidheen & Bodur [39], improvement in EDS was noted with modafinil after adenotonsillectomy for severe OSA in a child with NT1.

## 3 Pathogenesis

In the 1920s, Constantin von Economo, a distinguished Viennese neurologist, systematically examined post-mortem brain lesions in individuals who had succumbed to encephalitis lethargica. He hypothesized that the lateral hypothalamus functions as a critical center for the origination of a primary wake-promoting signal.

Orexin-A and orexin-B, identified as endogenous peptide ligands targeting orphan G-protein-coupled receptors (OX1 and OX2 receptors) by T. Sakurai et al., in 1998 [40], intricately modulate various physiological processes such as sleep, energy homeostasis, respiratory function, and reward-seeking behaviors. The activity of orexin neurons is subjected to regulatory influences, wherein glucose and leptin (the satiety hormone) exert inhibitory effects, while ghrelin (the hunger hormone) stimulates their activity. Notably, selective ablation of orexin cells results in diminished energy expenditure, ultimately contributing to the development of obesity, as elucidated by Williams and Burdakov [41]. While predominantly expressed in the lateral hypothalamic area of the brain, orexin peptides have also been identified in peripheral tissues, as documented by Heinonen and colleagues [42].

The relatively small population of orexin neurons, numbering in the thousands, extensively projects envelop nearly every region of the brain, excluding the cerebellum. These projections notably impact the reticular activating system's pivotal components and the attention-promoting regions of the cortex, thereby augmenting arousal, a phenomenon integral to fight-or-flight responses. Orexin firing is subject to inhibition by other arousal systems, such as noradrenaline and serotonin, suggesting the existence of a negative feedback loop.

Furthermore, impulses originating from the lateral hypothalamus exert a persistent facilitatory influence on the respiratory center, projecting specifically to the pre-Botzinger region of the rostral ventrolateral medulla and to phrenic motoneurons, as expounded by Williams and Burdakov [41]. In mouse animal models, the optogenetic activation of the circuit connecting the lateral hypothalamus to the pre-Botzinger complex induced sighing and tachypnea, while its pharmacologic inhibition suppressed both responses [43]. Overall, findings suggest a role of the circuit on ventilation, but its implication in sleep-related obstructive events remains to be determined. Physiologically, orexin neurons demonstrate a depolarization response and increased electrical activity under conditions of physiological acidosis, while alkalosis induces hyperpolarization and inhibits orexin cell firing. The orexinergic system actively stimulates the ventilatory response to CO2. Orexin knockout mice exhibit diminished cardiovascular and respiratory responses, which can be partially restored through orexin administration. Conversely, the selective orexin-1 receptor antagonist SB-334867 reduces the hypercapnic response in wild-type mice, as evidenced by Deng and colleagues in 2007 [44]. Spinieli et al. [45] observed a reduced ventilatory and behavioral response to hypoxia in rats undergoing an orexin receptor 1 and 2 blockade with suvorexant; these data suggest that orexin participate in the peripheral chemoreflex.

A study conducted by Han et al. [46] revealed a blunted hypoxic responsiveness in individuals with NT1 and healthy carriers of the HLA DQB1∗0602 allele compared to non-carriers, providing additional insights into the complex interplay of the orexinergic system in respiratory and cardiovascular regulation.

There is a proposed mechanism suggesting that orexin activation of the genioglossus muscle results in the dilation of upper airways, and consequently, the loss of orexin in NT1 may facilitate OSA. Conversely, the possibility exists that OSA may contribute to the development of narcolepsy through long-term damage to the orexin systems [25,47]. In addition, it was proposed that intermittent hypoxia, as provoked by recurrent apneas, could determine a disruption of the immune system, due to an increased T-helper 17 to regulatory T cells ratio, to an increase in interleukin 4 and other inflammatory citochines [48], and to an increase in diversity and clonotypes of T-cell receptors [49]. By mean of these immune processes, intermittent hypoxia has the potential to trigger an autoimmune damage affecting the orexin neurons [50,51], in line with multiple lines of evidence on the role of T-cell activity imbalance in the pathogenesis of NT [52,53].

Contrary to concerns about the potential respiratory side effects of orexin-receptor antagonists, commonly used in insomnia treatment, clinical trials and daily experiences suggest that they do not significantly impact breathing regulation. No relevant effects on AHI, respiratory disturbance index (RDI), or oxygen saturation were detected for laborexant [54,55] and daridorexant [56], even in patients with chronic pulmonary disease [57] or OSA [58]. Additionally, experimental molecules in this category showed no significant respiratory side effects [59].

Conflicting evidence surrounds plasma orexin levels in OSA patients, with some studies reporting lower levels compared to non-OSA groups [60], while others indicate an increase in plasma orexin-A levels in OSA subjects [61]. Further complexity is introduced by studies showing elevated orexin-A levels in OSA patients experiencing EDS [62]. Conflicting evidence arises from a study analyzing CSF orexin levels in severe OSA subjects, which found no significant differences compared to controls and NT2 patients [63].

In addition, impact of effective therapies for OSA, such as CPAP and adenotonsillectomy, on orexin levels in patients remains unclear [64]. On the contrary, studies on young obese adults undergoing bariatric surgery revealed a significant increase in plasma orexin levels, accompanied by improvements in clinical symptoms, a reduction in AHI, arousal index, and daytime sleepiness [65].

Metabolic syndrome and obesity represent another relevant line of evidence concerning the pathogenesis of OSA and NT1, and could account for the association of the two disorders. Orexin modulates growth hormone [66] and cortisol secretion [67], upregulates leptin [68], downregulates prolactin and improves insulin sensitivity [69]. Therefore, orexin deficiency affects endocrine function, leading to a reduced food-specific satiety [70], to an increased food intake and to a metabolic dysregulation [69,71], which in turns can provoke weight gain and diabetes. Neural correlates of the dysregulated food intake in NT1 were studied, and associated with decreased dorsal medial prefrontal cortex responses during general executive control and enhanced ventral medial prefrontal cortex responses during food-driven attention [72]. These findings could account for the strong association between obesity and NT1. However, the complex interplay of the abovementioned hormones and their link to obesity in NT1 remains to be elucidated, since patients with NT1 did not demonstrate a reduced metabolic rate [73], neither a reduced baseline level of ghrelin and leptin [74] compared to healthy controls.

NT1-related weight gain provokes a deposition of fat in the tongue and in the tissue surrounding the pharynx, increasing the upper airways collapsibility and potentially causing OSA [75].

Also, the other way around, OSA is a main risk factor for metabolic syndrome. The association was explained based on a mechanism of intermittent hypoxia, which provokes an increased sympathetic activation with impaired blood pressure, an increased hepatic glucose output, and insulin resistance through adipose tissue inflammation, pancreatic β-cell dysfunction and hyperlipidemia. Consequent obesity can increase the risk of NT1 [76].

## 4 Treatment of EDS in narcolepsy with comorbid OSA

EDS has been identified as a significant detriment to the quality of life, as evidenced by the Europe 2016–2017 National Health and Wellness Survey, which assessed the impact of sleepiness on the quality of life of individuals with various sleep disorders. Patients with OSA (n = 2277) narcolepsy (n = 48), or a combination of both conditions (n = 23) exhibited a substantial decrease in quality-of-life scores compared to individuals with non-pathological sleepiness [77]. This underscores the crucial need for effective management of EDS associated with OSA and/or narcolepsy.

The guidelines of the European Sleep Research Society on the management of narcolepsy indicates pharmacological and non-pharmacological approaches as effective on multiple symptoms dimensions. For excessive daytime sleepiness in adults scheduled naps, modafinil, pitolisant, sodium oxybate (SO), solriamfetol, methylphenidate and amphetamine derivatives are indicated; in children the same interventions except for pitolisant and solriamfetol are also recommended. For cataplexy antidepressants as clomipramine and venlafaxine are indicated in both adults and children, while SO and pitolisant in adults only [78].

Despite the use of CPAP for OSA, a surprising finding reveals that 38 % of patients using CPAP for more than 7 h still exhibited objective sleepiness, as demonstrated by Weaver and colleagues [79]. Additionally, some patients, despite achieving adequate adherence to CPAP, may remain undertreated for rapid eye movement (REM)-predominant OSA, contributing to persistent EDS. The consequences of OSA, including chronic sleep deprivation and hypoxia-reoxygenation, may result in persistent adverse effects on wake-activating neurons, as suggested by Javaheri & Javaheri [80].

The efficacy of stimulants in treating EDS in narcoleptic patients with comorbid OSA has not been rigorously studied through randomized controlled trials (RCTs), but evidence from distinct groups suggests that modafinil, commonly used in narcolepsy monotherapy, can improve both objective and subjective sleepiness in patients with OSA experiencing refractory sleepiness despite compliant CPAP use [80]. Pitolisant, a selective histamine H3 receptor antagonist/inverse agonist, has gained approval for alleviating symptoms of EDS in both NT1 and OSA based on several clinical trials [81,82]. A meta-analysis pooling data from four RCTs indicated that pitolisant significantly reduced EDS, increased mean sleep latency, and improved quality of life scores without significant adverse effects, except for insomnia [83]. Solriamfetol, a dopamine and norepinephrine reuptake inhibitor, received approval in 2019 for treating EDS in OSA and narcolepsy. It demonstrated efficacy in TONES studies and has advantages such as reduced abuse risk, renal excretion, and a favorable drug interaction profile compared to other medications [81,84]. Moreover, solriamfetol demonstrated a favorable effect on body weight, in patients with NT1 or OSA, with a dose dependent pattern [85]. However, notable adverse effects include headaches, anxiety, decreased appetite, and nausea. Solriamfetol should be used cautiously in certain patient populations, including those with cardiovascular instability, a history of bipolar disorder or psychosis, and those taking monoamine oxidase inhibitors [80]. An intriguing study revealed that individuals with NT1 did not experience worsening daytime sleepiness with the development of concomitant OSA [14]. Furthermore, stable nasal CPAP treatment did not lead to improvements in sleep latency during MSLT in these patients [86]. Moreover, another study found that CPAP therapy did not yield a significant improvement in EDS for most patients with NT1 and OSA, implying that OSA might not play a substantial role in exacerbating the severity of EDS in narcolepsy [30].

## 5 The controversial respiratory effect of sodium oxybate for treatment of NT1

The precise mode of action of SO remains uncertain, with suggested mechanisms including an increase in serotonin turnover, interaction with opioid systems, and potential agonism of gamma-aminobutyric acid B (GABA-B) receptors, as posited by Alshaikh and colleagues [87]. However, concerns have been raised regarding the impact of SO on co-existing sleep-related breathing disorders, particularly OSA, despite contradictory findings in the literature.

An improvement in central sleep apnea (CSA) was documented in a case report by Mamelak & Webster [88], where a patient with narcolepsy and central sleep apnea showed clear amelioration following SO treatment. Conversely, two cases involving patients with NT1 and comorbid heart diseases reported acute deleterious effects on breathing, leading to OSA. In one case, the respiratory event returned to baseline after SO withdrawal, while the other case necessitated CPAP use to normalize sleep breathing [89]. Interestingly, an opposite effect was observed in a 64-year-old man with OSA and narcolepsy, where SO administration resulted in a significant reduction of AHI from 32.3 to 13.2/hr [90]. However, in two other cases of narcolepsy with refractory cataplexy, SO use was associated with the occurrence of severe OSA, requiring CPAP therapy, in order to avoid discontinuation of SO [87].

In a case study involving patients with OSA, the acute effects of SO on sleep-disordered breathing were investigated. The administration of 9g/night sodium oxybate did not worsen OSA, but changes in central apnea and significant oxygen desaturations were observed in some patients, suggesting potential adverse effects on breathing [91]. However, the duration of desaturations was brief and clinically insignificant in most cases. Two additional investigations did not confirm the acute and deleterious effects of short-term use of 4.5g/night SO on patients with OSA concerning AHI and oxygen saturation [92,93]. Remarkably, the use of high doses of SO in OSA has not been extensively studied and warrants caution. It is emphasized that if a narcoleptic patient with comorbid sleep apnea or heart disease requires SO, administration should be conducted under polysomnographic control to monitor potential effects on sleep-disordered breathing.

In an adult patient with comorbid NT1, OSA and REM sleep behavior disorder (RBD) with concomitant onset, SO exerted an effect on muscle activity during REM sleep, improving RBD symptoms. However, SO also provoked and increase in the periodic limb movement (PLM) index and in nocturnal moaning when the patient was not using CPAP [94]. A favorable effect of SO was also described in both idiopathic [95] and Parkinson disease related [96] RBD. In contrast with these reports, a long-term study on 23 patients with NT1 treated with SO described 2 cases of RBD, possibly induced by the medication at a dose of 4.5g [97] indicating that the effect of SO is controversial also on RBD.

A beneficial effect of SO on OSA could be mediated by its favorable effect on the body mass index BMI. In patients with NT1, SO was consistently determining a weight decreases from obese to overweight or normal weight, and from overweight to normal weight, both in pediatric [[98], [99], [100]] and adult [101] populations. An increased lipolysis was advocated as the SO mechanism of action in this respect [102].

## 6 Discussion

The prevalence of OSA in patients affected by NT1 range between 24.8 % and 51.4 %, compared to a a range between 10 and 30 % in general population. These results corroborate the hypothesis that NT1 increase the risk to develop OSA, although the literature data are not conclusive, considering the relatively small sample size of the studies, the heterogeneity in diagnosis, assessment, and severity of OSA.

More in details, the studies included in the present review had some limitations: the AHI threshold to diagnose OSA was of 5/h^32,33,36^, 10/h^30^, 15/h^31^ or 1/h^37^, and it was not explicit in three studies [29,34,35]. Four studies [[29], [30], [31], [32]] had a retrospective design, while a prospective longitudinal one would have been optimal. Notwithstanding the limits of these review, we recommend to screen OSA in patients with NT1, over time, since the risk increase with aging, also taking into account the multiple cardiovascular risks in relation to comorbid obesity and to chronic treatment with stimulants.

In addition, literature data on comorbid OSA and narcolepsy in children are scarce, and it is not possible to have any conclusive results.

In any case, this association could be underpinned by the role of orexin in hypercapnic-hypoxic responses. The deficiency in orexin is supposed to contribute to the manifestation of obstructive events during sleep. Likewise, the prevalent coexistence of obesity in both OSA and NT1 implies a plausible connection. Although obesity may offer a partial explanation for this association, unresolved questions demand further exploration. In the context of NT1, the pronounced correlation with OSA might be elucidated by the active participation of orexin in the hypercapnic-hypoxic response, with its deficiency potentially contributing to obstructive events during sleep. In contrast, the association between OSA and narcolepsy type 2 is less clearly delineated.

There is limited information available regarding the efficacy of stimulants in patients with narcolepsy who either have comorbid OSA or residual EDS after OSA treatment. These gaps in knowledge necessitate further exploration. In adult patients diagnosed with OSA and EDS, or those experiencing persistent EDS despite receiving appropriate CPAP therapy, narcolepsy screening should be considered. The intricate overlap of symptoms emphasizes the significance of adopting a comprehensive and nuanced diagnostic approach. This involves a meticulous clinical evaluation and when warranted, additional testing to ensure accurate differentiation between these conditions. In the absence of established guidelines administering effective treatment for OSA is recommended before establishing a definitive diagnosis of narcolepsy. Furthermore, if NT1 is suspected, assessing CSF orexin levels may offer benefits in situations where the results of the MSLT could potentially yield false negatives, particularly in patients with OSA, experiencing persistent EDS, and using medications that suppress REM sleep.

## Funding

“This research received no external funding”

## Institutional review board statement

“Not applicable”

## Informed consent statement

“Not applicable”

## CRediT authorship contribution statement

**Silvia Miano:** Writing – review & editing, Writing – original draft, Project administration, Formal analysis, Conceptualization. **Leila Kheirandish-Gozal:** Writing – review & editing, Validation, Supervision. **Marco De Pieri:** Writing – review & editing, Writing – original draft, Methodology, Conceptualization.

## Declaration of competing interest

The authors declare that they have no known competing financial interests or personal relationships that could have appeared to influence the work reported in this paper.
